# Skeletal health in *DYRK1A* syndrome

**DOI:** 10.3389/fnins.2024.1462893

**Published:** 2024-09-06

**Authors:** Elysabeth D. Otte, Randall J. Roper

**Affiliations:** Department of Biology, Indiana University Indianapolis, Indianapolis, IN, United States

**Keywords:** haploinsufficiency, gene dosage, mouse models, Down syndrome, Hsa21

## Abstract

*DYRK1A* syndrome results from a reduction in copy number of the *DYRK1A* gene, which resides on human chromosome 21 (Hsa21). *DYRK1A* has been implicated in the development of cognitive phenotypes associated with many genetic disorders, including Down syndrome (DS) and Alzheimer’s disease (AD). Additionally, overexpression of *DYRK1A* in DS has been implicated in the development of abnormal skeletal phenotypes in these individuals. Analyses of mouse models with *Dyrk1a* dosage imbalance (overexpression and underexpression) show skeletal deficits and abnormalities. Normalization of *Dyrk1a* copy number in an otherwise trisomic animal rescues some skeletal health parameters, and reduction of *Dyrk1a* copy number in an otherwise euploid (control) animal results in altered skeletal health measurements, including reduced bone mineral density (BMD) in the femur, mandible, and skull. However, little research has been conducted thus far on the implications of *DYRK1A* reduction on human skeletal health, specifically in individuals with *DYRK1A* syndrome. This review highlights the skeletal phenotypes of individuals with *DYRK1A* syndrome, as well as in murine models with reduced *Dyrk1a* copy number, and provides potential pathways altered by a reduction of *DYRK1A* copy number, which may impact skeletal health and phenotypes in these individuals. Understanding how decreased expression of *DYRK1A* in individuals with *DYRK1A* syndrome impacts bone health may increase awareness of skeletal traits and assist in the development of therapies to improve quality of life for these individuals.

## 1 Introduction

### 1.1 *DYRK1A* gene dosage imbalance and *DYRK1A* syndrome

Dual-specificity tyrosine phosphorylation-regulated kinase 1A (*DYRK1A*) is a highly conserved gene located on human chromosome 21 (Hsa21) with known roles in neuronal and skeletal development. Variations in *DYRK1A* copy number lead to phenotypes associated with several developmental disorders, suggesting that a critical gene-dosage balance of *DYRK1A* is required for proper early development. *DYRK1A* syndrome, also known as *DYRK1A* haploinsufficiency syndrome and Intellectual developmental disorder, autosomal dominant 7 (MRD7) (OMIM:614104) is generally caused by the presence of a pathogenic variant of *DYRK1A*, that reduces the functional copy number of *DYRK1A* from two to one, and likely decreases DYRK1A protein levels (Arranz et al., 2019). This disorder is presently characterized by intellectual disability, the development of autism spectrum disorder (ASD), and behavioral problems. Individuals with *DYRK1A* syndrome also display non-cognitive phenotypes including altered facies, intrauterine growth restriction, short stature, and reduced skeletal growth. The potential skeletal abnormalities associated with *DYRK1A* syndrome have not been well characterized, but insights from the consequences of dosage imbalance of *DYRK1A* and information from mouse models with altered *Dyrk1a* copy number may provide a basis for further examining the skeletal health of individuals with *DYRK1A* syndrome.

The purpose of this review is to examine existing case reports and studies detailing skeletal abnormalities in individuals with *DYRK1A* syndrome, as well as to highlight skeletal parameters in *Dyrk1a* haploinsufficient mouse models. Our objective is to call attention to potential skeletal phenotypes affecting individuals with *DYRK1A* syndrome, and provide potential molecular pathways leading to these abnormalities, so that therapies may be found to improve quality of life for these individuals.

## 2 Characteristics of the *DYRK1A* gene and gene conservation between species

**Figure 1:**
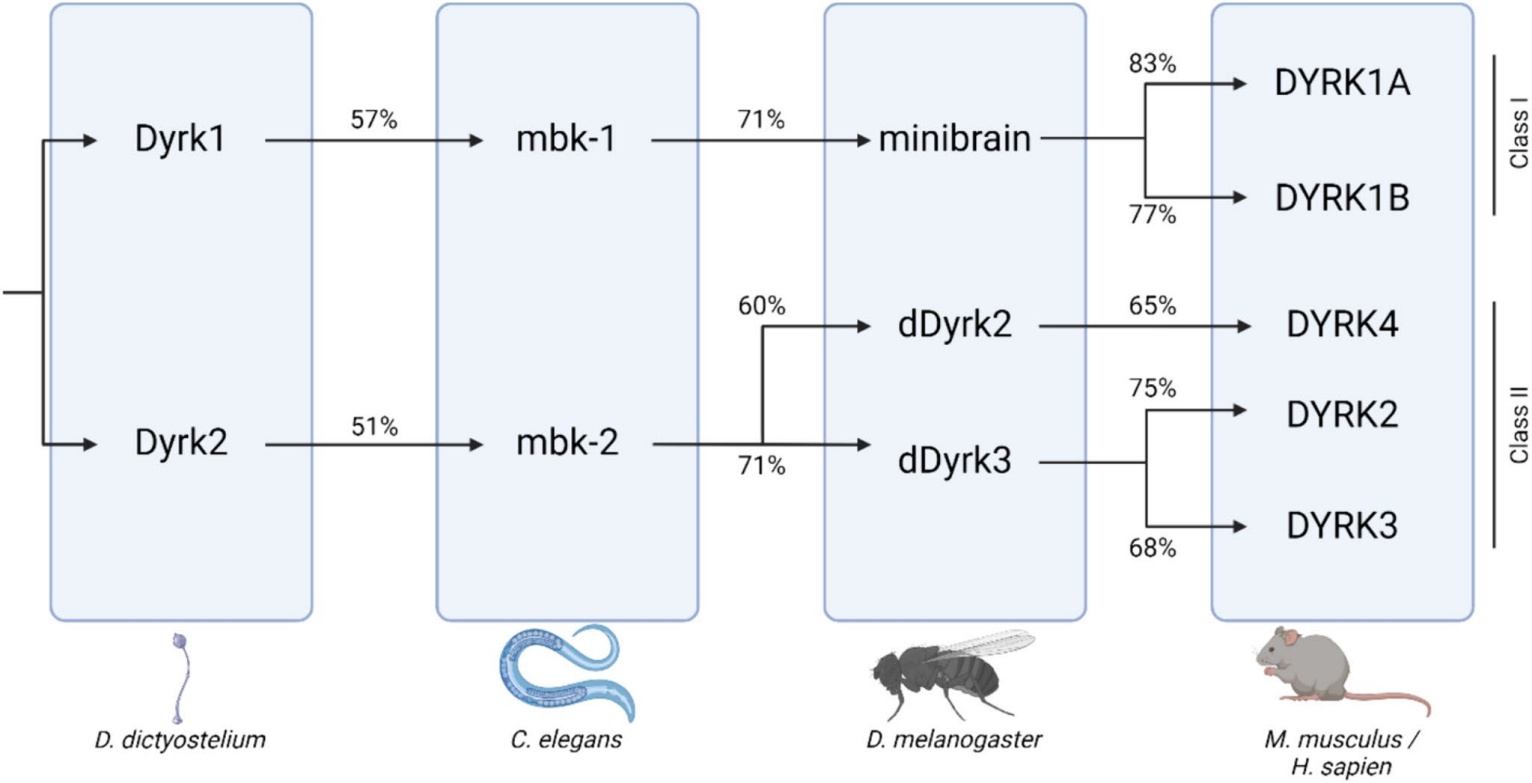
DYRK protein kinase family tree across species, including *D. Dictyostelium*, *C. elegans*, *D. melanogaster*, and mammals. Percent orthology between species shown beneath connecting arrows [adapted from Deboever et al. (2022); created with BioRender].

*DYRK1A* gene dosage imbalance has been associated with multiple disorders affecting both cognitive ability and skeletal health, including Down syndrome (DS), Alzheimer’s disease (AD), Parkinson’s disease (PD), and Huntington disease (HD) (Deboever et al., 2022). *DYRK1A* is a member of the DYRK family of kinases. DYRK protein kinases are activated by autophosphorylation on a tyrosine residue in the activation loop, which catalyzes the phosphorylation of exogenous serine and threonine residues. DYRK proteins are evolutionally conserved, and orthologs of *DYRK1A* are found in animal models including mouse (*Mus musculus*--*Dyrk1a*), fruit fly (*Drosophila melanogaster*--Mnb), and nematode (*Caenorhabditis elegans*--*mbk-1*) (Figure 1) (Alvarez et al., 2007; Rammohan et al., 2022). Null mutations in or removal of both copies of the *DYRK1A* gene are embryonic lethal in humans and mammalian models, but non-mammalian knockout models, including *C. elegans* strains without *mbk-1*, are viable. In other experimental systems, models containing either one or three copies of the gene are utilized to understand more about the effects of *DYRK1A* dosage imbalance. Together, data examining the phenotypes associated with both increased and decreased *Dyrk1a* expression indicate a dosage-dependent role of *Dyrk1a* on neural and skeletal system development and function.

### 2.1 Expression of *DYRK1A* in multiple tissues

*DYRK1A* is ubiquitously expressed in all human tissues and is essential for neurogenesis, cell cycle regulation, and synaptic plasticity. DYRK1A/MNB is involved in the phosphorylation of microtubule dynamics, which help to regulate dendritic patterning and neuronal function (Ori-McKenney et al., 2016; Deboever et al., 2022). The effects of mammalian *Dyrk1a* are found throughout embryonic development and continue into neonatal neurogenesis, and high levels of *DYRK1A* have been detected in the adult cerebellum (Deboever et al., 2022). *Dyrk1a* has also been implicated in the development of craniofacial features. Alterations in face size and neural crest cells in animal models with *Dyrk1a* deficiency and overexpression may be explained by increased cell death and decreased cellular proliferation in cellular precursors to the face (McElyea et al., 2016; Johnson et al., 2024).

## 3 *DYRK1A* syndrome genetics and phenotypes

Illustrating the dosage-dependent properties of *DYRK1A*, *DYRK1A* syndrome is an autosomal dominant disorder often caused by a loss-of-function mutation within the *DYRK1A* gene. Findings in individuals with *DYRK1A* syndrome suggest a potential heterozygous *de novo* pathogenic variant in *DYRK1A*, leading to the development of this diverse disorder (van Bon et al., 2015). Over 120 disease-related variants have been reported in *DYRK1A*, the majority of which are small deletion mutations, missense mutations, nonsense mutations, and small insertion mutations. The majority of missense pathogenic variants of the *DYRK1A* gene are located in the kinase domain, leading to a disruption of the catalytic domain and kinase activity of the protein (van Bon et al., 2015). In addition, some studies suggest that phenotypes associated with *DYRK1A* syndrome may be compounded by mutations in nearby genes, including potassium inwardly rectifying channel subfamily J member 6 (*KCNJ6*), also located nearby on Hsa21. Mutations in *KCNJ6* have been associated with the development of Keppen-Lubinsky syndrome, leading to severe developmental delay, facial dysmorphism, intellectual disability, and seizures (Valetto et al., 2012).

### 3.1 Cognitive and behavioral phenotypes of *DYRK1A* syndrome

Individuals with *DYRK1A* syndrome display cognitive and behavioral phenotypes including the development of ASD, anxiety, epilepsy, and sleep disturbances (Ji et al., 2015; van Bon et al., 2015; van Bon et al., 2016). *DYRK1A* syndrome is believed to account for up to 0.5% of individuals with intellectual disability, and studies have found that *DYRK1A* is one of the most frequent *de novo* mutated genes in ASD (Arbones et al., 2019). The cognitive phenotypes associated with this disorder have been the most widely characterized, as intellectual disability and developmental delay are some of the most common symptoms present in these individuals (van Bon et al., 2015).

### 3.2 Other (non-cognitive) phenotypes in *DYRK1A* syndrome including skeletal malformations

Other phenotypes commonly associated with individuals with *DYRK1A* syndrome include intrauterine growth restriction (IUGR), microcephaly, hypertonia, urogenital abnormalities, characteristic facial features, micrognathia, feeding difficulties, short stature, and musculoskeletal anomalies (Earl et al., 2017; Meissner et al., 2020; Huang et al., 2023). These phenotypes are often noted perinatally, and many persist after birth. Slow head growth rates into adulthood result in an increasingly severe microcephaly phenotype. Altered skull development and formation may be a result of not only neuronal impacts of reduced *DYRK1A* expression but may also be a product of altered growth of the skull in these individuals. Facial dysmorphisms have been reported in approximately 98% of published cases of *DYRK1A* syndrome, supporting the hypothesis of the dosage-dependent role of *DYRK1A* in craniofacial development (Earl et al., 2017). Dental anomalies, including widely spaced teeth, delayed primary dentition, and supernumerary teeth have also been reported in individuals with *DYRK1A* syndrome, along with typical facial gestalt (Desai, 1997; van Bon et al., 2016; Huang et al., 2023).

In comparison to the cognitive and behavioral phenotypes associated with the disorder, skeletal and growth abnormalities are largely unexplored and reflect limited research examining the potential impacts of *DYRK1A* syndrome on skeletal health. One study evaluating the stature and growth of individuals with *DYRK1A* syndrome found that over half of individuals surveyed had a short stature and low birth weight (Lanvin et al., 2024). Short stature may be due to prenatal and/or early childhood development, while low birth weight often persists later into life. Skeletal abnormalities amongst these individuals commonly impact the hands and feet and associated bones. Several case studies of individuals with *DYRK1A* syndrome have described contractures of the ankles, feet, and hands, as well as cavovarus foot deformities, tibial osteochondrosis, high arched feet, long hallux, and arachnodactyly (Earl et al., 2017; Huang et al., 2023). Additionally, approximately 10% of individuals with *DYRK1A* haploinsufficiency develop musculoskeletal abnormalities, including scoliosis, kyphosis, and pectus excavatum. These musculoskeletal phenotypes target the spine, sternum, and ribs, which may be indicative of the effects of *DYRK1A* haploinsufficiency on skeletal development and growth. Further understanding the dosage-dependent role of *DYRK1A* in skeletal development, and skull formation and growth may help to elucidate how these phenotypes may be closely associated.

## 4 *DYRK1A* dosage imbalance (overexpression) is linked to many phenotypes in Trisomy 21 or Down syndrome (DS)

Individuals with DS may have up to 300 protein-coding genes triplicated, including *DYRK1A.* This dosage imbalance of *DYRK1A* is present in almost all individuals with Trisomy 21, leading to DS (OMIM:190685). This imbalance, in combination with other triplicated genes, has been implicated in the development of many phenotypes associated with DS (Blazek et al., 2015; Deboever et al., 2022; Redhead et al., 2023; Lana-Elola et al., 2024). Individuals with DS often present with alterations in multiple organ systems, including congenital heart defects, musculoskeletal abnormalities, cognitive deficits, respiratory issues, and intestinal problems (Antonarakis et al., 2020). Mouse models of DS have been used to verify the role of *DYRK1A* in the development of these phenotypes. For example, in the Ts65Dn DS mouse model, with about half of the genes homologous to Hsa21 in three copies, increased DYRK1A protein levels in neural stem cells of the cerebellar cortex lengthens the G1 phase and reduce neuronal production, supporting the role of *DYRK1A* in the development of cognitive phenotypes associated with DS (Najas et al., 2015). Like those with *DYRK1A* haploinsufficiency, individuals with DS often present with intellectual disability, along with craniofacial abnormalities, and musculoskeletal deficits, potentially due to the roles of DYRK1A in bone formation, osteoclastogenesis, neurogenesis, and synaptogenesis (Lee et al., 2009; Park and Chung, 2013).

### 4.1 Skeletal abnormalities associated with *DYRK1A* overexpression

Skeletal abnormalities, including small body size, reduced bone mineral density (BMD) and weaker bone strength are often characteristic phenotypes of individuals with Trisomy 21. Deficits in skeletal microarchitecture often lead to the development of osteopenia or osteoporosis in individuals with DS (Angelopoulou et al., 1999). Additionally, these individuals often show delayed skeletal age in relation to chronological age, and reach skeletal maturation earlier than individuals without DS, due to a pronounced period of growth during a shorter period of skeletal maturation (de Moraes et al., 2008). Many of these characteristics have been, at least in part, attributed the gene-dose imbalance of *DYRK1A* in these individuals (Arron et al., 2006; Thomas and Roper, 2021; Sloan et al., 2023).

**Figure 2:**
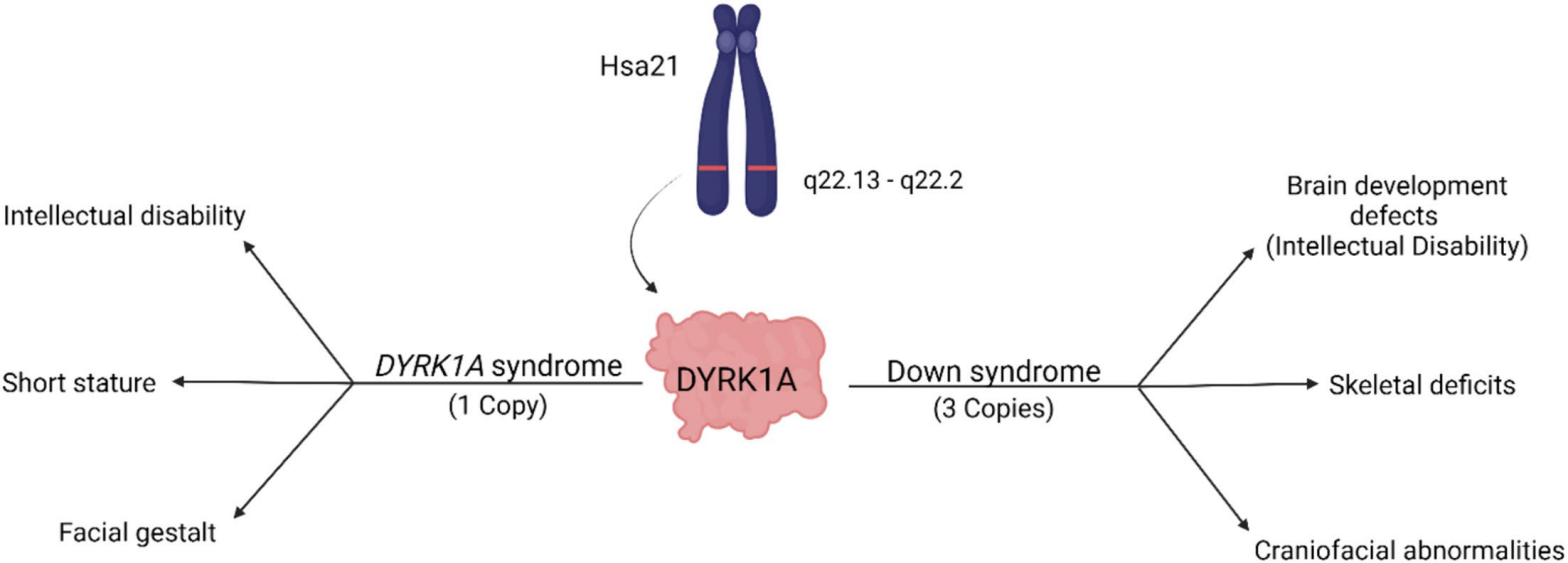
Dosage-dependent effects of the *DYRK1A* gene. Overexpression of *DYRK1A* is associated with Down syndrome phenotypes, while underexpression of *DYRK1A* results in the development of *DYRK1A* syndrome. Both disorders present with phenotypes affecting cognition, craniofacial development, and skeletal health [adapted from Laham et al. (2021); Created with BioRender].

A reduction of *DYRK1A* to one copy (*DYRK1A* haploinsufficiency) or an increase to three copies (Trisomy 21), may lead to similar tissues that are affected and comparable phenotypes (Figure 2 and Table 1), thus illustrating the effect of *DYRK1A* dosage imbalance (underexpression or overexpression) in humans. The impact of *DYRK1A* dosage imbalance may be better understood in experimental models where *Dyrk1a* dosage can be manipulated and studied. From our studies of DS mouse models (*Dyrk1a* overexpression) and *Dyrk1a* haploinsufficiency mouse models (*Dyrk1a* underexpression), *DYRK1A* dosage imbalance may lead to skeletal alterations that need to be better characterized in individuals with *DYRK1A* haploinsufficiency syndrome.

**Table 1:** Skeletal phenotypes associated with haploinsufficiency (1 copy – *DYRK1A* syndrome) or trisomy (3 copies – Down syndrome) of *DYRK1A*.

*DYRK1A* haploinsufficiency syndrome		Trisomy 21 or Down syndrome	
1 copy *DYRK1A*		3 copies *DYRK1A*	
Phenotypes	Reference	Phenotypes	Reference
Short stature	Meissner et al. (2020)	Short stature	Rarick et al. (1966)
Facial gestalt and craniofacial dysmorphology	van Bon et al. (2016)	Craniofacial dysmorphology	Richtsmeier et al. (2000)
Microcephaly	Ji et al. (2015)	Microcephaly	Torrado et al. (1991) and Angelopoulou et al. (1999)
Reduced skeletal growth	Lanvin et al. (2024)	Earlier completion of bone maturation, decreased bone mineral density	Angelopoulou et al. (1999) and de Moraes et al. (2008)
Intrauterine growth restriction	Earl et al. (2017)	Fetal growth restriction	Yao et al. (2020)
Dental abnormalities	van Bon et al. (2016)	Dental abnormalities	Desai (1997)
Tibial osteochondrosis	Huang et al. (2023)	Increased risk of osteoporosis and osteopenia	Angelopoulou et al. (1999)

**Figure 3:**
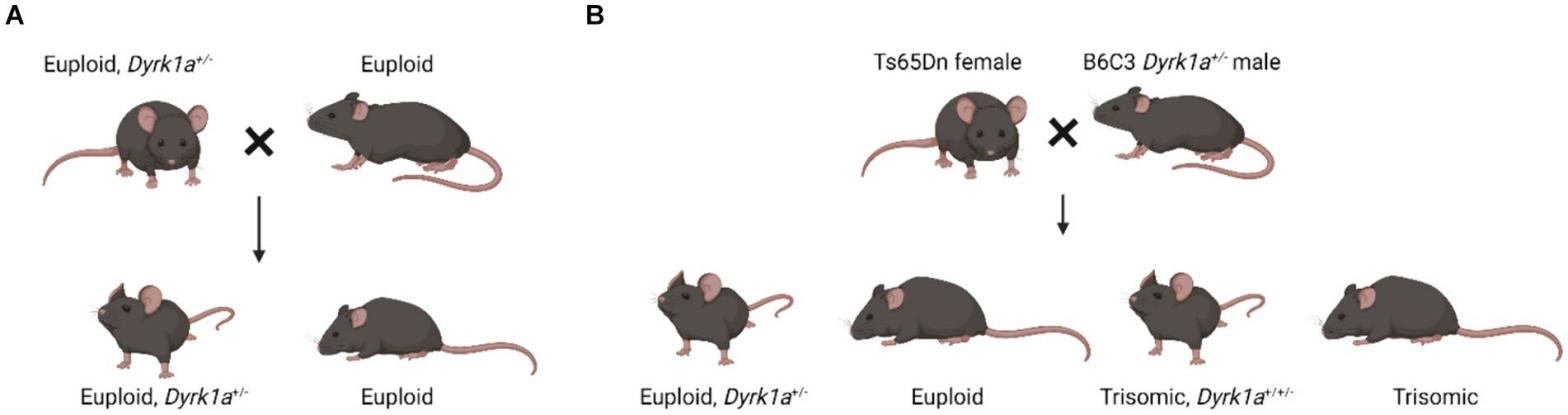
Breeding schematics used to generate euploid, *Dyrk1a*^+/−^ mice (Fotaki et al., 2002; LaCombe et al., 2024; Created with BioRender).

Observations in mouse models with dosage imbalance of *Dyrk1a* lead to a hypothesis that a reduction of *Dyrk1a,* as in *DYRK1A* syndrome, and an increase of *Dyrk1a,* as in Trisomy 21, both cause skeletal deficits, providing a foundation for the study of these phenotypes in humans. Male and female mice transgenic for extra human *DYRK1A* exhibited reduced trabecular bone measures, but did not show changes in cortical thickness (Lee et al., 2009). Additionally, these mice had reduced osteoclastogenesis and defective osteoblastogenesis. Mouse models of *DYRK1A* haploinsufficiency have been created by mutating exons 3, 5, or 6 of the *Dyrk1a* gene. These mice have either been bred to wild-type mice, and approximately half of the progeny will carry a single copy of *Dyrk1a,* or these mice have been bred to trisomic mice where approximately a quarter of the mice may carry a single copy of *Dyrk1a* (Figure 3).

## 5 Haploinsufficiency of *Dyrk1a* in mouse models causes cognitive phenotypes similar to *DYRK1A* syndrome

Mouse models with only one functional copy of *Dyrk1a* show phenotypes similar to those identified in humans with *DYRK1A* syndrome. *Dyrk1a* heterozygous knockout mice containing a frameshift mutation in exon 3 of *Dyrk1a* have been used to examine the cognitive and behavioral phenotypes of the disorder, and have shown that haploinsufficient mice display cognitive flexibility deficits, as well as sociability and communication impairments (Raveau et al., 2018). Mice containing targeted disruptions of *Dyrk1a* gene in exons 5 and 6 also present with neurological traits including hyperthermia-induced seizures and defective social interactions, closely mimicking the phenotypes observed in humans with *DYRK1A* haploinsufficiency (Arranz et al., 2019). These *Dyrk1a* haploinsufficient mice also display reductions in hippocampally-dependent memory tasks and impairments in novel object recognition tasks when compared to euploid controls (Arque et al., 2008).

Many of these cognitive phenotypes are believed to be tied to glial cell abnormalities and impaired neocortical circuit development caused by *Dyrk1a* dosage imbalance. *Dyrk1a* heterozygous mice compared to control mice of the same background have a decreased brain size and have been found to present with increased astrogliogenesis in the neocortex and delays in cortical oligodendrocyte progenitor cell production (Fotaki et al., 2002). Additionally, axonal conductivity impairment is believed to be altered in these animals, caused by decreased myelination and thinner axons in the corpus callosum (Pijuan et al., 2022). These haploinsufficient mice displayed abnormal proportions of excitatory and inhibitory neocortical neurons, caused by altered excitatory neuron production during early and mid-corticogenesis, and present with smaller, less branched pyramidal cells in the neocortex (Benavides-Piccione et al., 2005; Arranz et al., 2019).

Neurological traits associated with *DYRK1A* imbalance may additionally be contributed to dopaminergic neuron imbalance caused by abnormal activity of the caspase9-dependent apoptotic pathway (Barallobre et al., 2014). Mice haploinsufficient for *Dyrk1a* display hypoactivity and altered gait, suggesting a role of *Dyrk1a* in the development of the neuromotor system, and which may be directly correlated with altered dopaminergic activity (Fotaki et al., 2004; Martinez de Lagran et al., 2007). Administration of dopaminergic antagonists resulted in increased seizure activity in wild-type animals that was not seen in *Dyrk1a* haploinsufficient animals, suggesting that *Dyrk1a* haploinsufficient mice may have altered self-regulatory mechanisms of dopamine neurons (Martinez de Lagran et al., 2007). Additional studies from our lab examining DS have yielded mice with *Dyrk1a* haploinsufficiency and have begun to examine the skeletal phenotypes of these animals.

### 5.1 Skeletal phenotypes seen in *Dyrk1a* haploinsufficient mice

Compared to the effects of triplication of *Dyrk1a* on skeletal health, there are far fewer studies examining the effects of *Dyrk1a* haploinsufficiency on skeletal health and development using mammalian models. Similar to individuals with *DYRK1A* haploinsufficiency, mouse models with one copy of *Dyrk1a* have been used to characterize more than cognitive and behavioral deficits; yet many growth and skeletal deficits have been observed in these mice. Our laboratory has utilized a *Dyrk1a*^+/−^ mouse with a mutation spanning exons 5 and 6 that shows a number of skeletal abnormalities (Fotaki et al., 2002; Arranz et al., 2019).

**Figure 4:**
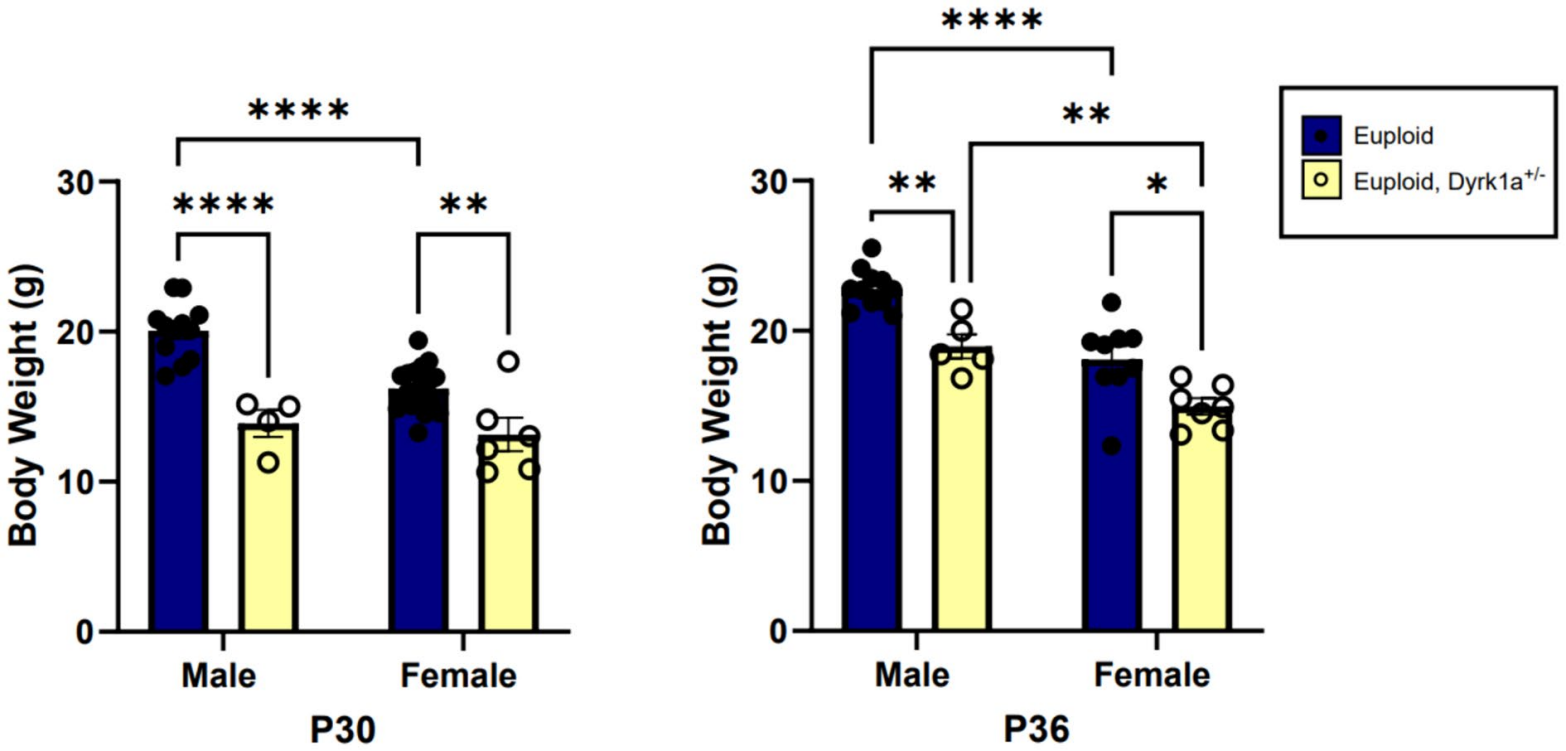
Body weight comparisons between euploid and euploid, *Dyrk1a*^+/−^ male and female animals at P30 and P36, highlighting reduced weight in both male and female euploid, *Dyrk1a*^+/−^ animals. Data analyzed using two-way ANOVA. Male: euploid (*n* = 12), euploid, *Dyrk1a*^+/−^ (*n* = 4); female: euploid (*n* = 19), euploid, *Dyrk1a*^+/−^ (*n* = 6). *p < 0.05, **p < 0.01, ****p < 0.0001. [Data from LaCombe et al. (2024)].

Male and female mouse models with only one copy of *Dyrk1a* exhibit smaller body mass as compared to control mice at postnatal day (P)30 and P36, which may be indicative of lower bone mass (Figure 4) (Fotaki et al., 2002; LaCombe et al., 2024). Some changes in cranial face and mandible but not cranial base or vault were observed in 6-week-old *Dyrk1a* haploinsufficient as compared to control mice (McElyea et al., 2016). Reduction of *Dyrk1a* copy number from conception in otherwise euploid 6-week-old male mice led to a significant decrease in mandible and skull BMD, along with reductions in femur BMD (Blazek et al., 2015). Otherwise normal male mice with *Dyrk1a* haploinsufficiency displayed a decrease in trabecular number, thickness, area, and perimeter in the 6-week-old (a time of bone formation roughly equivalent to the skeletal age of humans under the age of 20) femur when compared with euploid littermates (Blazek et al., 2015). Measurement of mechanical properties of the same animals showed decreases in ultimate force and stiffness in femurs from *Dyrk1a* haploinsufficient animals when compared to euploid mice. The skeletal measurements in *Dyrk1a* haploinsufficient mice were similar to those found in trisomic or DS model mice, potentially due to alterations in osteoclastogenesis caused by *Dyrk1a* gene dosage imbalance, and these skeletal phenotypes were normalized in trisomic animals with a normalized copy number of *Dyrk1a* (Blazek et al., 2015). The skeletal abnormalities found in *Dyrk1a* haploinsufficient mice may portend similar skeletal deficiencies in humans with *DYRK1A* haploinsufficiency.

### 5.2 Sexual dimorphism in *Dyrk1a* haploinsufficient mice

**Figure 5:**
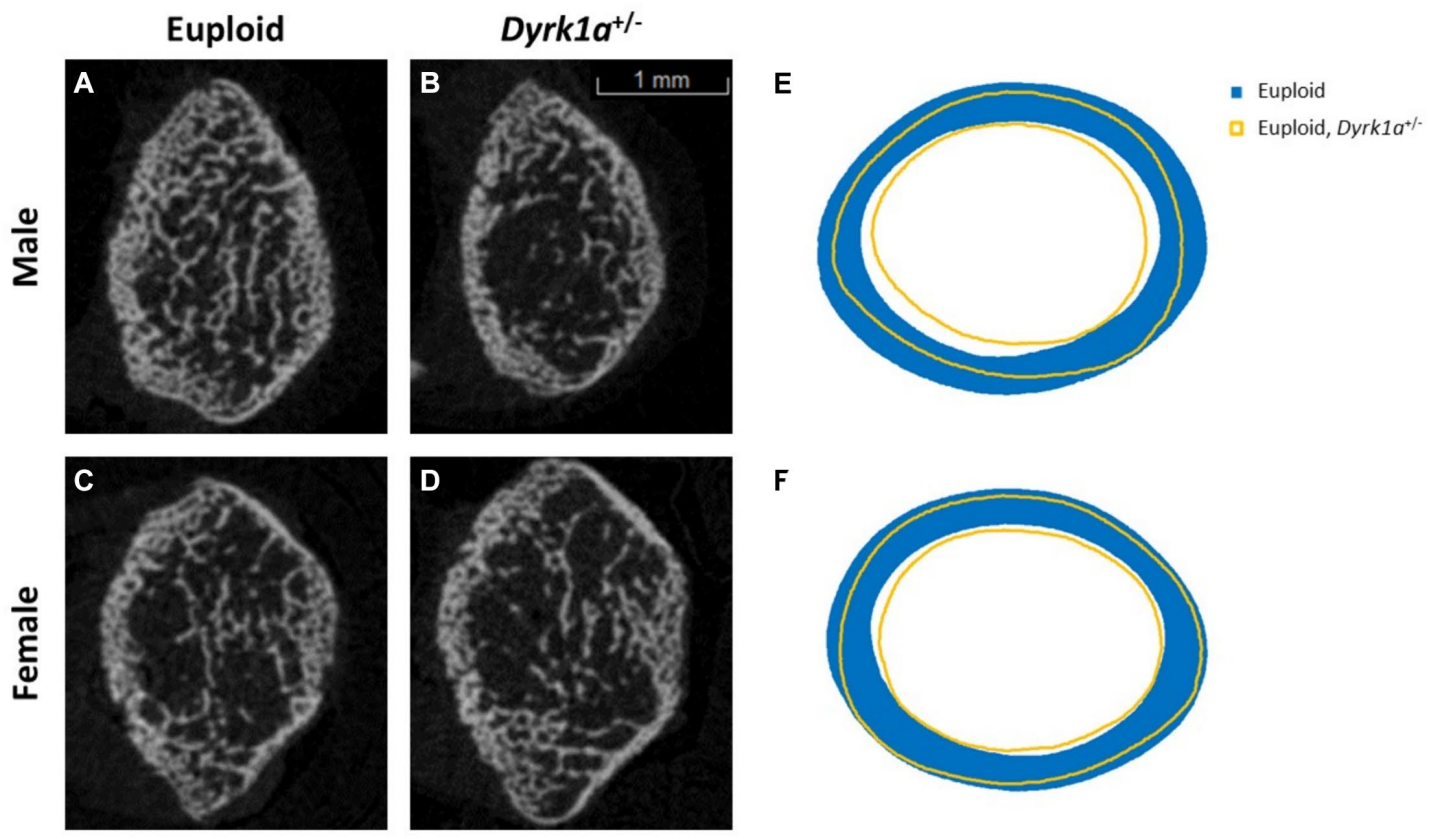
Trabecular and cortical bone are affected by *Dyrk1a* copy number at P30. Representative images of trabecular structure taken at P30 of euploid males **(A)**, euploid, *Dyrk1a*^+/−^ males **(B)**, euploid females **(C)**, and euploid, *Dyrk1a*^+/−^ females **(D)** as revealed by μCT analysis. Radar graphs of average periosteal and endocortical perimeter measurements of male **(E)** and female **(F)** euploid and euploid, *Dyrk1a*^+/−^ animals [Data from LaCombe et al. (2024)].

**Figure 6:**
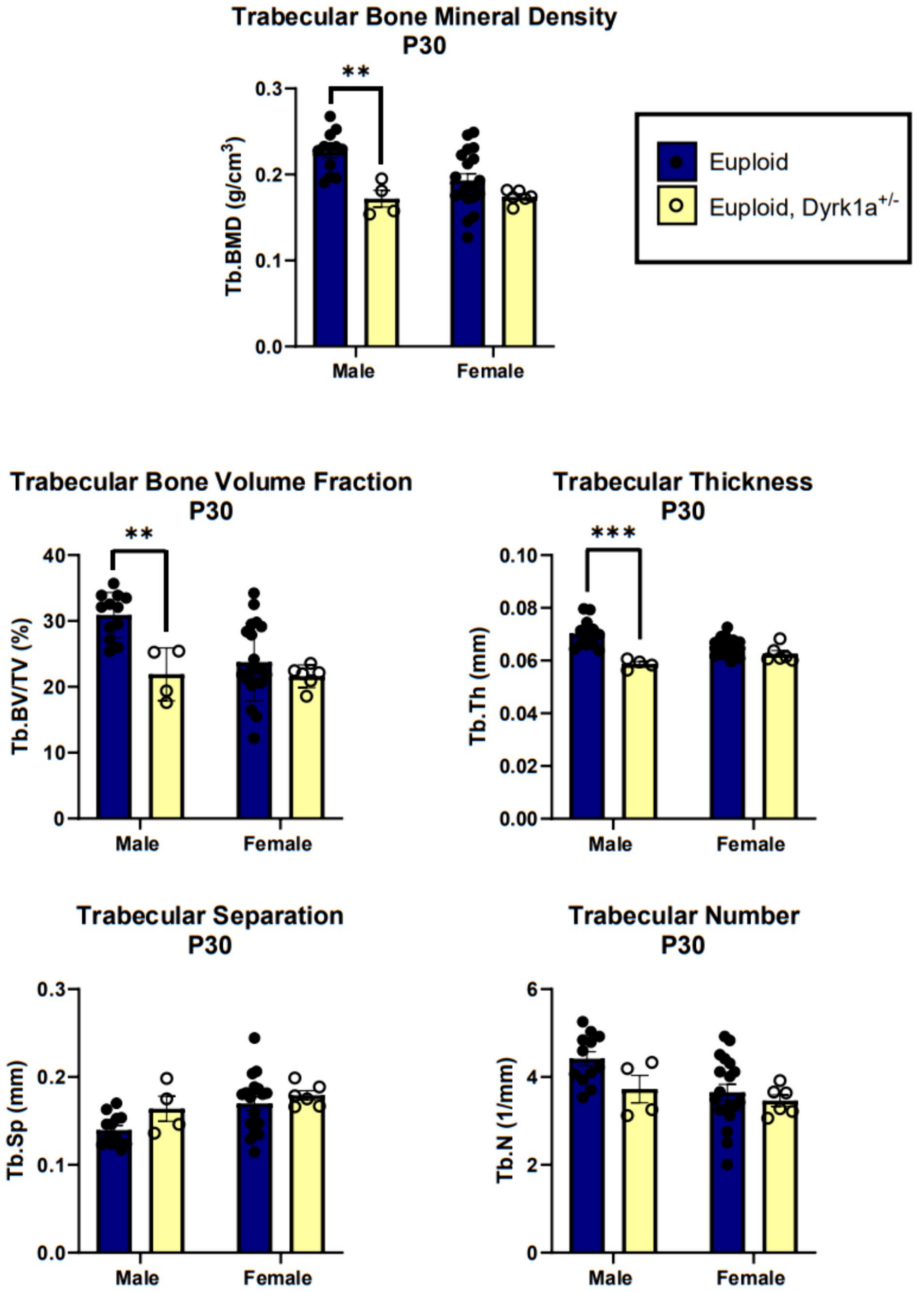
Trabecular bone measurements of male and female euploid and euploid, *Dyrk1a*^+/−^ animals at P30. Data analyzed using one-way ANOVA and Tukey/Games-Howell *post hoc* analysis. Male: euploid (*n* = 12), euploid, *Dyrk1a*^+/−^ (*n* = 4); female: euploid (*n* = 19), euploid, *Dyrk1a*^+/−^ (*n* = 6). **p < 0.01, ***p < 0.001. [Data from LaCombe et al. (2024)].

**Figure 7:**
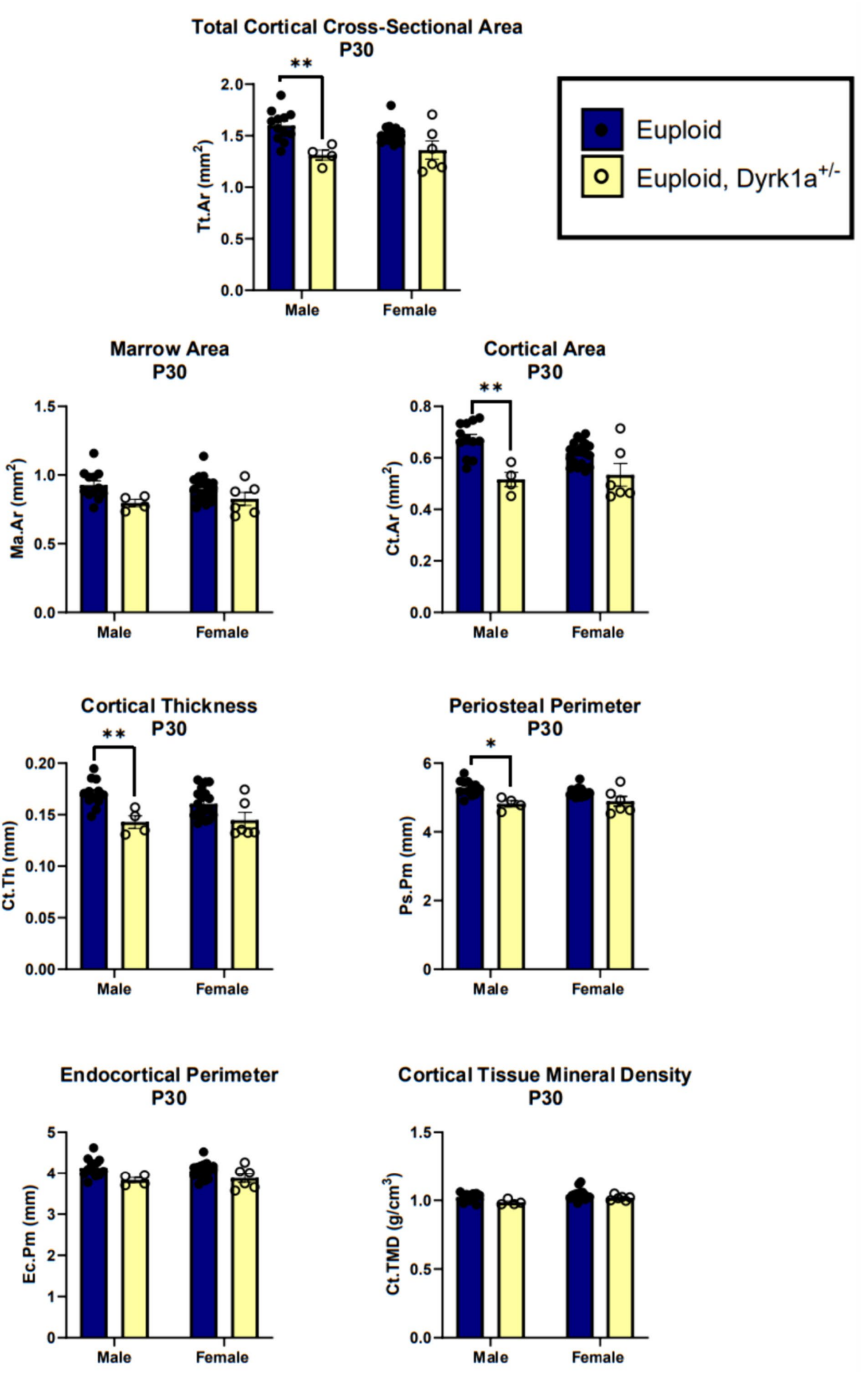
Cortical bone measurements of male and female euploid and euploid, *Dyrk1a*^+/−^ animals at P30. Data analyzed using one-way ANOVA and Tukey/Games-Howell *post hoc* analysis. Male: euploid (*n* = 12), euploid, *Dyrk1a*^+/−^ (*n* = 4); female: euploid (*n* = 19), euploid, *Dyrk1a*^+/−^ (*n* = 6). *p < 0.05, **p < 0.01. [Data from LaCombe et al. (2024)].

Additionally, we have shown that mice with a single copy of *Dyrk1a* may result in sexually dimorphic skeletal phenotypes, with skeletal deficits occurring earlier in male mice. In the femur of *Dyrk1a* haploinsufficient mice at P30 and P36, both trabecular and cortical bone are more severely affected in male than in female mice (Figure 5) (Thomas et al., 2021; LaCombe et al., 2024). In *Dyrk1a* haploinsufficient mice at P30 (an early time of bone formation), male animals haploinsufficient for *Dyrk1a* displayed reductions in trabecular BMD, bone volume fraction, and thickness in the femur when compared to euploid mice (Figure 6). *Dyrk1a* haploinsufficent male mice also displayed reductions in cortical cross-sectional area, thickness, periosteal perimeter, and section modulus (Figure 7). Female *Dyrk1a* haploinsufficient animals did not exhibit significant trabecular or cortical deficits at P30. At P36, femurs from male mice with only one copy of *Dyrk1a* displayed reductions in trabecular thickness and section modulus. Once again, female mice with one copy of *Dyrk1a* did not display significant trabecular or cortical deficits at P36 (LaCombe et al., 2024). These data emphasize both potential confounding effects of sex and age on skeletal deficits in *Dyrk1a* haploinsufficent animals, and similar sex-and temporally-specific skeletal abnormalities may be seen in humans with *DYRK1A* syndrome. For example, female individuals with *DYRK1A* syndrome may exhibit bone deficits later than males. Additionally, there may be periodic normalizations of some skeletal phenotypes throughout development (LaCombe et al., 2024; Lamantia et al., 2024). Knowing these sex-and temporally-specific details may provide a timeline for identifying skeletal phenotypes associated with *DYRK1A* syndrome and a “target” timeframe for possible therapeutic interventions.

### 5.3 Potential effects of DYRK1A inhibitors on skeletal health

The use of DYRK1A inhibitors has been a topic of research for several years as a potential pharmacotherapeutic treatment for DS phenotypes. The use of these inhibitors in DS mouse models has also shown the possible effects of these DYRK1A inhibitors in euploid (normal) littermate controls. Treatment of 3-week-old euploid animals with a purported DYRK1A inhibitor, epigallocatechin-3-gallate (EGCG), resulted in a significant increase in femoral bone volume as well as trabecular number and thickness when compared to trisomic Ts65Dn animals. Treatment of euploid animals with 9 mg/kg/day EGCG resulted in significantly increased percent bone volume, but did not alter the mechanical properties or BMD of the femur (Blazek et al., 2015). As treatment with EGCG increased, male euploid animals exhibited decreased cortical thickness (Jamal et al., 2022). Treatment with 50 mg/kg/day EGCG of male euploid animals showed slight reductions in cortical bone phenotypes. These data further support the dosage-dependent hypothesis regarding *Dyrk1a* and suggest that pharmaceutical reduction of DYRK1A activity may alter skeletal formation and health. The effects of EGCG in DS animal models and control mice suggested that this inhibitor may affect products of *Dyrk1a* or other trisomic genes in the Ts65Dn mouse model and negatively impact bone structure and function. Additional studies examining how EGCG may impact skeletal health have found that different supplements exhibit different effects on skeletal measures, with some resulting in improvement, and others being detrimental (Abeysekera et al., 2016). Treatments using DYRK1A inhibitors must carefully be administered at proper levels so that characteristics of *DYRK1A* syndrome, especially in bone, are not induced (Stringer et al., 2017).

### 5.4 Possible mechanisms of *DYRK1A* haploinsufficiency causing skeletal deficits

The effects of reduced *DYRK1A* likely involve many molecular pathways to produce skeletal and other deficits. DYRK1A acts on the PI3K/AKT/mTOR pathway via modulation of brain-derived neurotrophic factor (BDNF) (Rammohan et al., 2022). BDNF has been suggested to play a role in fracture healing and bone remodeling (Xue et al., 2021). Additionally, the PI3K/AKT/mTOR and NFATc pathways play a role in skeletal development and homeostasis (Arron et al., 2006; Chen, 2018).

The skeletal system contains a widely distributed neurovascular system that undergoes active remodeling during bone regeneration and repair. Nearly all the sensory nerves in bone express neurotrophic receptor tyrosine kinase type 1 (TrkA), which is a receptor for nerve growth factor (NGF). NGF activation of TrkA plays a role in the activation of osteogenic cues leading to bone formation through the Wnt/β-catenin pathway. *DYRK1A* overexpression has negative effects on NGF signal transduction *in vitro*, suggesting another potential pathway through which *DYRK1A* dosage may influence skeletal growth and health (Stefos et al., 2013; Tomlinson et al., 2017).

DYRK1A also interacts with the RE1-silencing transcription factor (REST), also known as the neuron restrictive silencing factor (NRSF), which acts as an inhibitor of cell proliferation and differentiation and has been suggested to be a regulator of early osteoblast differentiation. REST is traditionally known as a master neuronal negative regulator transcription factor that functions to maintain transcriptional silence of neuronal genes, though recent research has begun to examine its effects outside of neuronal maintenance. Studies have found that REST regulates early osteoblast differentiation via an unknown pathway. Alterations in *Dyrk1a* expression have been found to modify *Rest/Nrsf* gene expression, which may in turn change osteoblast differentiation and lead to skeletal abnormalities in individuals with altered levels of *DYRK1A* expression (Lepagnol-Bestel et al., 2009; Liu et al., 2015).

Additionally, DYRK1A has been found to be a promoter of fibroblast-like synoviocytes (FLS), which play a role in cartilage/bone destruction in rheumatoid arthritis (RA) via activation of the ERK/MAPK pathway (Guo et al., 2018). More recent data has shown that conditional reduction of *Dyrk1a* in murine chondrocytes results in increased progression of osteoarthritis (OA), suggesting a protective effect of *Dyrk1a* in disease progression, potentially via modulation of the EGFR-ERK signaling in articular chondrocytes (Liu et al., 2023). These influences on the ERK signaling pathway may alter bone growth and development through modulation of osteoblast differentiation (Kim et al., 2022). We hypothesize that by critically examining *Dyrk1a* haploinsufficent mice, we will gain a better understanding of how reduced *Dyrk1a* copy number leads to skeletal deficits at the molecular level, including specific *Dyrk1a* mutations that may lead to particular skeletal deficits.

## 6 Discussion

Though no studies have concentrated on skeletal health parameters in individuals with *DYRK1A* syndrome, reports of individuals with *DYRK1A* haploinsufficiency have shown the development of several phenotypes that may indicate skeletal deficits, including intrauterine growth abnormalities, short stature, craniofacial and dental abnormalities, and developmental abnormalities in hand, foot, and ankle. Skeletal abnormalities are a common phenotype seen in individuals with intellectual disabilities, potentially partially due to decreased activity levels in these individuals. However, examination of the molecular connections that may cause both cognitive and skeletal phenotypes is worth considering. Mouse model studies with overexpression and underexpression of *Dyrk1a* provide foundational evidence that *DYRK1A* copy number dosage imbalance alters skeletal development and health, showing that both increased and decreased gene dosage results in skeletal health deficits. Additional studies designed to examine skeletal development in mouse models of *DYRK1A* syndrome should be conducted to provide evidence of the impacts of underexpressed *DYRK1A* on bone health, especially concentrating on molecular mechanisms. These data would provide additional avenues for the development of therapies targeting *DYRK1A* syndrome, to increase the quality of life of these individuals. Current early intervention treatments recommended for individuals diagnosed with *DYRK1A* haploinsufficiency include physical therapy to increase mobility and modulate risk for scoliosis and other musculoskeletal symptoms (van Bon et al., 2015). These early treatments may additionally modulate skeletal phenotypes that have yet to be uncovered, as chronic exercise is a known inducer of bone growth and remodeling. Current evidence supports the role of *DYRK1A* in skeletal formation and health and suggests that skeletal phenotypes should be examined in individuals with *DYRK1A* syndrome. From the data presented, these skeletal deficits may appear in males earlier than females with *DYRK1A* haploinsufficiency. Molecular pathways influenced by *DYRK1A* that may lead to both cognitive and skeletal phenotypes must also be studied, and the effects of a gene-dosage imbalance of *DYRK1A* need to be fully characterized. Further understanding the roles of this dosage sensitive gene in the development of multiple organ systems may aid in the potential development of pharmacotherapies to treat the symptoms associated with both DS and *DYRK1A* syndrome.
